# Non-invasive MRI quantification of cerebrospinal fluid dynamics in amyotrophic lateral sclerosis patients

**DOI:** 10.1186/s12987-019-0164-3

**Published:** 2020-01-21

**Authors:** Lucas R. Sass, Mohammadreza Khani, Jacob Romm, Marianne Schmid Daners, Kyle McCain, Tavara Freeman, Gregory T. Carter, Douglas L. Weeks, Brian Petersen, Jason Aldred, Dena Wingett, Bryn A. Martin

**Affiliations:** 1 Neurophysiological Imaging and Modeling Laboratory, University of Idaho, 875 Perimeter Dr. MS1122, Moscow, ID 83844 USA; 2 University of Washington School of Medicine, 1959 NE Pacific St, Seattle, WA 98195 USA; 3 Product Development Group Zurich, Department of Mechanical and Process Engineering, ETH Zurich, Zurich, Switzerland; 4 St. Luke’s Rehabilitation Institute, 711 South Cowley St., Spokane, WA 99202 USA; 5 Inland Imaging PS and LLC, 801 South Stevens St., Spokane, WA 99204 USA; 6 Selkirk Neurology, 610 South Sherman St. #201, Spokane, WA 99202 USA; 7 Inland Imaging LLC, 801 South Stevens St., Spokane, WA 99204 USA; 8 Biological Engineering, University of Idaho, 875 Perimeter Dr. MS0904, Moscow, ID 83844-0904 USA

**Keywords:** Cerebrospinal fluid, Amyotrophic lateral sclerosis, Magnetic resonance imaging, Intrathecal drug delivery, Spinal cord

## Abstract

**Background:**

Developing novel therapeutic agents to treat amyotrophic lateral sclerosis (ALS) has been difficult due to multifactorial pathophysiologic processes at work. Intrathecal drug administration shows promise due to close proximity of cerebrospinal fluid (CSF) to affected tissues. Development of effective intrathecal pharmaceuticals will rely on accurate models of how drugs are dispersed in the CSF. Therefore, a method to quantify these dynamics and a characterization of differences across disease states is needed.

**Methods:**

Complete intrathecal 3D CSF geometry and CSF flow velocities at six axial locations in the spinal canal were collected by T2-weighted and phase-contrast MRI, respectively. Scans were completed for eight people with ALS and ten healthy controls. Manual segmentation of the spinal subarachnoid space was performed and coupled with an interpolated model of CSF flow within the spinal canal. Geometric and hydrodynamic parameters were then generated at 1 mm slice intervals along the entire spine. Temporal analysis of the waveform spectral content and feature points was also completed.

**Results:**

Comparison of ALS and control groups revealed a reduction in CSF flow magnitude and increased flow propagation velocities in the ALS cohort. Other differences in spectral harmonic content and geometric comparisons may support an overall decrease in intrathecal compliance in the ALS group. Notably, there was a high degree of variability between cases, with one ALS patient displaying nearly zero CSF flow along the entire spinal canal.

**Conclusion:**

While our sample size limits statistical confidence about the differences observed in this study, it was possible to measure and quantify inter-individual and cohort variability in a non-invasive manner. Our study also shows the potential for MRI based measurements of CSF geometry and flow to provide information about  the hydrodynamic environment of the spinal subarachnoid space. These dynamics may be studied further to understand the behavior of CSF solute transport in healthy and diseased states.

## Background

Amyotrophic lateral sclerosis (ALS), also known as Lou Gehrig’s disease, is a devastating neurological disorder of predominately sporadic origin [1] that leads to severe disability and death. While the majority of cases are sporadic, approximately 10% show familial inheritance [2]. ALS results in the loss of upper and lower motor neurons from the motor cortex, brainstem and spinal cord. Neurodegeneration in ALS typically advances in a sequential fashion to the point of phrenic nerve involvement resulting in failure of respiratory effort and death before degenerative changes are seen elsewhere [3]. ALS affects approximately 3.9 in 100,000 people within the United States [4] with approximately equal occurrence worldwide and does not appear to be linked with environmental toxins. Studies indicate that ALS incidence is approximately 1.8 times greater in males than females for unknown reasons [5].

A current challenge in identifying treatments for ALS is finding reliable measures of efficacy. Historically, survivability is one of the main metrics used in this determination [6]. Through examination of the CSF system we aim to add to the understanding of ALS pathophysiology and potentially provide another avenue of diagnosing or monitoring the disease in a quantitative manner. Developing novel therapeutic agents to treat ALS has also been difficult because of the high degree of disease heterogeneity and multifactorial pathophysiologic processes at work [6, 7]. A growing area of research surrounding ALS treatment is intrathecal (IT) drug administration. Researchers have investigated safety, tolerability, and pharmacodynamics of IT injection for a range of ALS therapeutics [6]. Additionally, filtration of cerebrospinal fluid (CSF) is actively being developed as a treatment for cryptococcal meningitis [8], subarachnoid hemorrhage [9] and has been used experimentally in ALS [10, 11].

To be effective, delivery of IT therapies rely on transport within CSF, movement of the drug across the meninges, transport along the perivascular spaces and finally absorption into CNS tissue. The use of in vivo measurements along with computer models of CSF solute transport could help maximize drug dispersion and help avoid toxicity. Magnetic resonance imaging (MRI) is an effective tool for non-invasively measuring CSF flow. Several studies have already used phase contrast MRI (PCMRI) to measure and reconstruct CSF flow dynamics in silico [12–14].

The goal of the present study was to characterize CSF flow dynamics and geometry in people with ALS compared to healthy controls. A few groups have made strides towards characterizing CSF dynamics in conditions such as Chiari malformation [15–21], Syringomyelia [20–22], and hydrocephalus [23], as well as investigation of CSF flow dynamics in people with ALS [24]. We expand on this characterization by providing a more complete analysis of the hydrodynamic environment. Consideration of hydrodynamics could aid in development of emerging therapeutics while also expanding the pathophysiologic understanding of this disease.

## Methods

Inclusion criteria for people with ALS were diagnosis of clinically suspected or definite ALS and able to tolerate the MRI scan without contraindications. Exclusion criteria included: presence of connective tissue disorder, previous history of cardiovascular disease, intracranial mass/deformity, CSF leak, spinal cord tethering, spina bifida, or myelomeningocele. Ultimately, eight participants with ALS were recruited from a regional adult population. The size of this population and restriction on travel compensation further limited recruitment of people with ALS in this pilot study. Ten healthy adult controls were also recruited based on the same exclusion criteria and the inclusion criterion of tolerance for the MRI scan without contraindications. Prior to each MRI scan, subject height, weight, waist circumference, heart rate, and blood pressure were recorded. MRI data collection was performed at Inland Imaging in Spokane, WA.

### MRI CSF flow measurement protocol

**Fig. 1:**
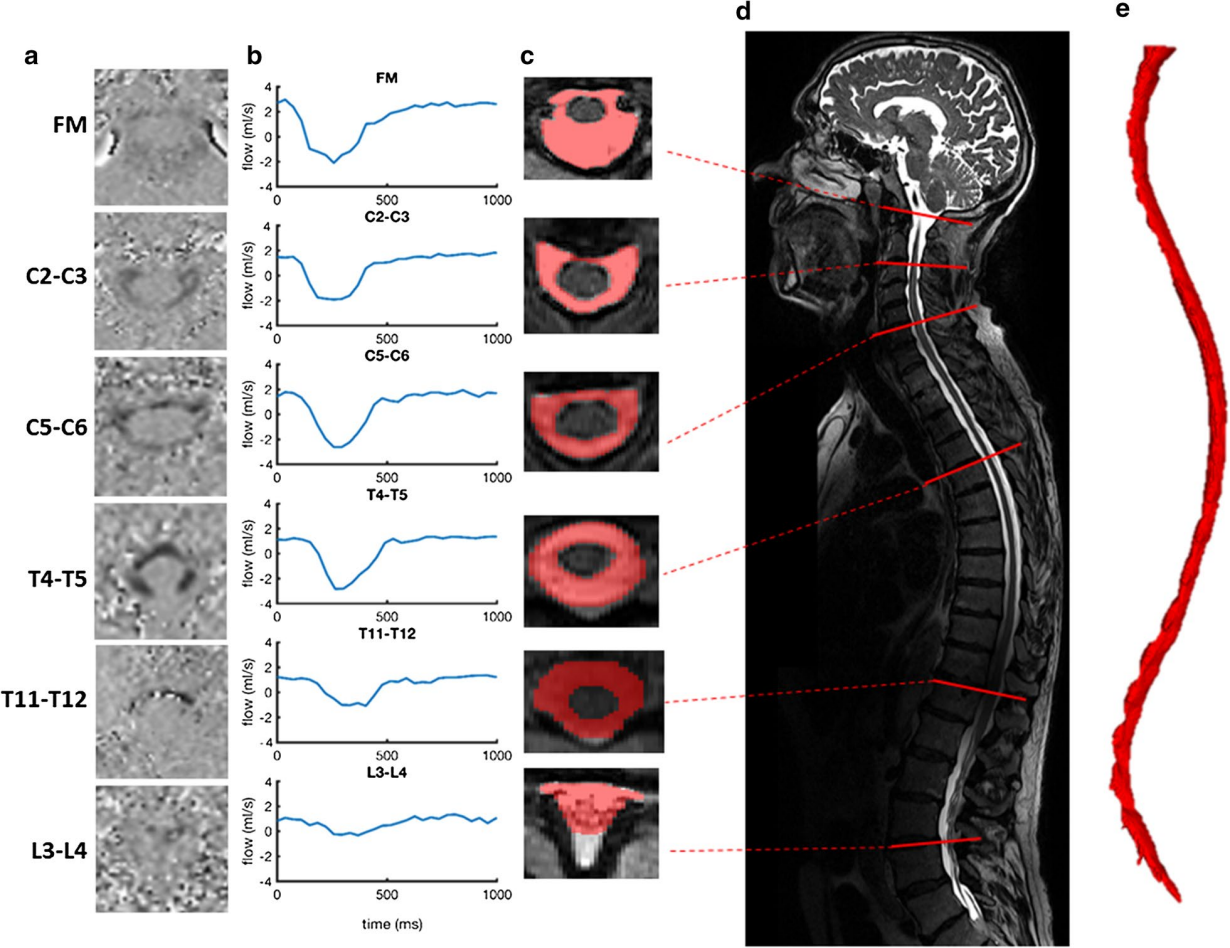
Subject specific example of CSF flow and geometric reconstruction for control 008. **a** Phase-contrast MRI at each of the six axial locations along the spine. **b** CSF flow rate based on PCMRI measurements collected at the FM, C2–C3, C5–C6, T4–T5, T11–T12, and L3–L4. **c** Axial view of semi-automatic contrast-based segmentation of T2-weighted MRI slices. **d** Full spine sagittal T2-weighted MR image including the position of axial segments of interest. **e** Final 3D geometric model of the SSS

MRI measurements were obtained on a Siemens 3T Skyra (Software version syngo MR E11, Siemens Corporation, Munich, Germany). Identical CSF flow measurements were taken for all subjects at six vertebral locations, Foramen Magnum (FM), C2–C3, C5–C6, T4–T5, T11–T12, and L3–L4 using PCMRI with retrospective gating from pulse oximetry for 30 cardiac phases (Fig. 1a). Slice thickness at each location was 5.0 mm with an in-plane isotropic resolution of 781 µm (~ 150 × 200 pixel FOV). Each slice was oriented perpendicular to the direction of CSF flow with slice plane aligned at the location of the vertebral disks (Fig. 1d). Values used for the flip angle, TR, TE, and VENC were 20°, 20.34, 6.69 and 10 cm/s, respectively. Total imaging time to collect all six slices was ~ 10 min.

### CSF flow quantification

The CSF flow rate, $Q_{CSF} \left( t \right)$, was calculated for each of the six spinal locations shown in Fig. 1d by importing the PCMRI data into MATLAB R2016b (The Mathworks Inc., Natick, MA, USA). $Q_{CSF} \left( t \right)$ was computed based on the numerical integration of individual pixel velocities over the CSF area ($A_{CSF}$) for an entire cardiac cycle: $Q_{CSF} \left( t \right) = \sum A_{voxel} \left[ {v_{voxel} \left( t \right)} \right]$, where $A_{voxel}$ is the in-plane area of one PCMRI voxel, and $v_{voxel}$ is the CSF velocity encoded in that voxel (Fig. 1b). The CSF waveform for the entire spinal cord (SC) was derived through interpolation of CSF flow between each of the six axial measurements. Methods were previously developed by our group for calculating CSF and cerebral blood flow rates [15, 25] as well as CSF flow interpolation [13, 26].

### MRI CSF space geometry protocol

A stack of high-resolution sagittal T2-weighted sampling perfection with application optimized contrasts using different flip angle evolution (SPACE) magnetic resonance (MR) images of the complete spinal subarachnoid space (SSS) anatomy was acquired for each subject (Fig. 1d). These images were acquired with 437 µm isotropic in-plane resolution with 800 µm slice thickness and spacing in three blocks (craniocervical, thoracic, and lumbosacral). Total imaging time for both MRI scan types was ~ 42 min.

### CSF space segmentation

**Fig. 2:**
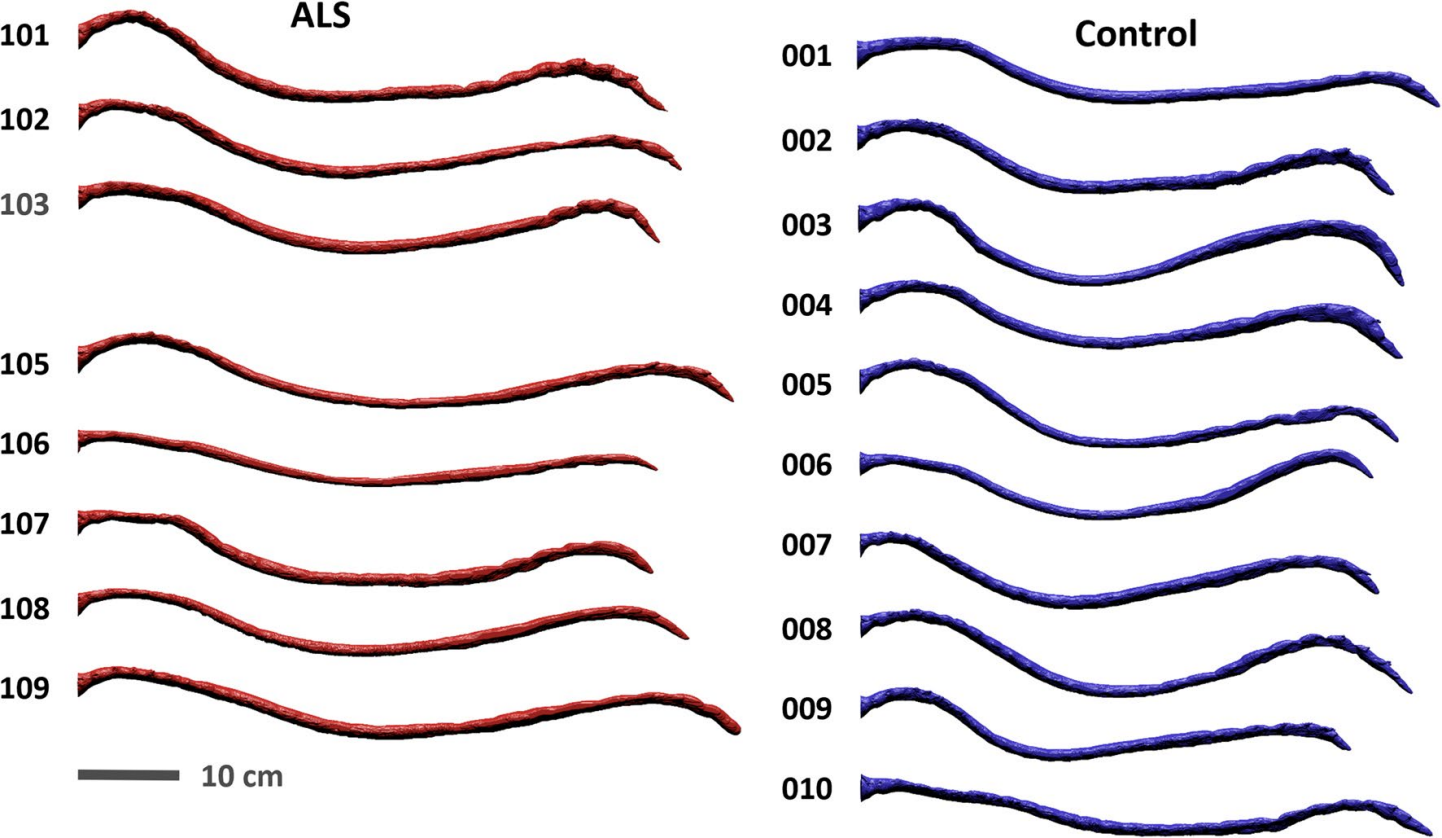
Geometric models of the spinal subarachnoid space for all subjects created by an expert operator based on T2-weighted MRI data segmentation. ALS subject 104 is not included as they withdrew from the study before collection of MRI data

The segmentation of MRI data was performed using the open-source program, ITK-SNAP (Version 3.4.0, University of Pennsylvania, USA). The MR image set for each spinal segment was manually reconstructed from an axial view with the semi-automatic contrast-based segmentation tool (Fig. 1c), as performed by our group in previous work [17]. Segmentation from the FM to the end of the dural sac was completed by one of two trained operators (Figs. 1e and 2). Anatomical fine structures such as SC nerve roots (except at the filum terminale) and denticulate ligaments were not possible to accurately visualize, given the MRI resolution with which the scans were collected. Consequently, these structures were not included in the segmentation (Fig. 1e).

### Geometric analysis

Similar to our previous studies, computational meshing was used to calculate the following geometric parameters along the spine [27]: First, the cross-sectional area of the SC, $A_{c}$, and region bounded by the dura, *A*_*d*_, were used to obtain the cross-sectional area of the SSS, $A_{cs} = \, A_{d} {-} \, A_{c}$. The hydraulic diameter for internal flow within a tube, $D_{H} = 4A_{cs} /P_{cs}$, was calculated based on the cross-sectional area and wetted perimeter, $P_{cs} = \, P_{d} + \, P_{c}$. Wetted perimeter is the sum of the SC, *P*_*c*_, and dura, *P*_*d*_, perimeter. A user-defined function was used to calculate each of these parameters in ANSYS FLUENT (Ver. 19.2, ANSYS inc, Canonsburg, PA, USA).

### Hydrodynamic analysis

Hydrodynamic environment was assessed at 1-mm slice intervals along the entire spine by Reynolds number based on the peak flow rate, and Womersley number based on hydraulic diameter. In calculating Reynolds number, $Re = \frac{{Q_{sys} D_{H} }}{{\nu A_{cs} }}$, $Q_{sys}$ is the temporal maximum of the local flow at each axial location along the spine obtained by interpolation from the experimental data. The kinematic viscosity of CSF, given by $\nu = \mu /\rho$, was assumed to be the same as water at body temperature. At peak systole, the presence of laminar flow along the spine was characterized using Reynolds number (Re < 2300) similar to previous studies in CSF mechanics [13, 17, 26]. The Womersley number, $\alpha = D_{H} \sqrt {\omega /\nu }$, was computed where ω is the angular velocity of the volumetric flow waveform with $\omega = 2/T$ and $\nu$ is the kinematic viscosity of CSF as defined above. The Womersley number can be used to characterize the ratio of unsteady inertial forces to viscous forces for the CSF of the SSS [28]. CSF pulse wave velocity ($PWV$) along the spine was quantified as a possible indicator of SSS compliance, as performed in our previous studies [13, 26]. In brief, a linear fit of the peak systolic flow arrival time along the spine was used to compute $PWV$, equal to the slope of the linear fit.

### CSF waveform analysis

For flow rate and frequency analysis, it was necessary to perform normalization and alignment of the flow data. CSF flow rate at each of the six axial measurement locations were offset-corrected such that the net flow rate corresponded to 0 mL/s. Average flow rate was calculated for the C2–C3 location [29]. Because of disparate temporal offsets introduced by the use of pulse oximetry in PCMRI phase gating, the steepest decent assessed at the C2–C3 location was used to align the data in time. This phase shift was done on a subject-specific basis with the time shift value calculated at the C2–C3 location applied across all locations. The data was then extended to 1280 ms during diastole and resampled at 10-ms intervals to avoid influencing the fast Fourier transformation (FFT) and to allow temporal comparison across all subjects. The cardiac cycle over all subjects was 956 ± 138 ms and the longest duration was 1264 ms. For the frequency analysis, the data at all six axial locations was spatially normalized by the average flow rate at the C2–C3 location with the goal to emphasize the flow patterns at all locations rather than to assess the individual flow rates. The frequency components of the FFT are expressed in harmonics (−). The data analysis and visualization were performed within MATLAB R2016b.

### Statistical analysis

Descriptive statistics were obtained for each parameter analyzed in terms of mean and standard deviation of values at each axial location for the ALS and control groups. Average values over the entire spine were also computed for each parameter along with the total value for parameters such as total spinal cord, dura and SSS volume. Statistical analysis was conducted in MATLAB R2016b. Feature points and the individual frequency components were statistically compared with a Mann–Whitney U test. Differences were considered significant at a p-value < 0.05.

## Results

The ALS group was comprised of 7 males and 1 female with average age of 56 ± 10 years. The control group included 6 males and 4 females averaging 59 ± 12 years of age. The tabulated results for all parameters quantified in the ALS group and corresponding values in the control group may be found in Table 1. MR images of healthy controls revealed no major abnormalities, such as degenerate disks or CSF stenoses that would be considered to affect CSF flow dynamics or geometry. One person with ALS (101) had a nonfunctioning IT pain medication pump and two ALS patients (106 and 108) had a history of spinal surgery (laminectomy and L4–S1 spinal fusion respectively). These subjects were included in our final ALS cohort as no anomalies were present in the SSS geometry. Another person with ALS (102) had near zero flow at all locations and was therefore excluded from the flow and hydrodynamic analyses (Figs. 4, 5, 6, 7). In the case of near zero flow, we confirmed that the PCMRI sequence triggered correctly by verification of pulsatile arterial and venous blood flow patterns to the brain visible in the imaging.

**Table 1:** Geometric and hydrodynamic results

Parameter	Control Avg.^a^ ± std.	ALS Avg.^a^ ± std.
Perimeter SC (cm)	1.87 ± 0.22	1.88 ± 0.26
Perimeter DM (cm)	5.66 ± 0.78	5.25 ± 0.83
Perimeter SSS (cm)	7.53 ± 0.89	7.13 ± 0.96
Cross-sectional area SC (cm^2^)	0.35 ± 0.07	0.35 ± 0.08
Cross-sectional area DM (cm^2^)	2.13 ± 0.54	1.89 ± 0.49
Cross-sectional area SSS (cm^2^)	1.78 ± 0.53	1.53 ± 0.48
Volume SC (V_c_) (cm^3^)	20.99 ± 2.55	22.53 ± 2.29
Volume DM (V_d_) (cm^3^)	129.19 ± 18.67	119.83 ± 21.74
Volume SSS (V_cs_) (cm^3^)	108.20 ± 18.67	97.30 ± 20.51
Surface area SC (cm^2^)	113.52 ± 7.67	119.37 ± 6.59
Surface area DM (cm^2^)	344.12 ± 27.30	333.54 ± 39.72
Surface area SSS (cm^2^)	457.64 ± 29.89	452.91 ± 43.82
Hydraulic diameter (cm)	0.96 ± 0.21	0.89 ± 0.21
Reynolds number (cm)	194.74 ± 80.75	209.37 ± 37.34
Womersley number (α)	12.71 ± 1.48	11.92 ± 1.51
U_sys_ (cm/s)	− 1.30 ± 0.34	− 1.29 ± 0.33
U_dia_ (cm/s)	0.76 ± 0.30	0.70 ± 0.28
Q_sys_ (cm^3^/s)	− 1.88 ± 0.62	− 1.58 ± 0.31
Q_dia_ (cm^3^/s)	1.06 ± 0.29	0.83 ± 0.17
PWV (cm/s)	347.41 ± 88.13	473.19 ± 162.04

*DM* dura matter, *PWV* pulse wave velocity, *SC* spinal cord, *SSS* spinal subarachnoid space, *Q*_*dia*_ average diastolic CSF flowrate, *Q*_*sys*_ average systolic CSF flowrate, *U*_*sys*_ average systolic CSF velocity, *U*_*dia*_ average diastolic CSF velocity

^a^Average values are based 1 mm slice intervals along the entire spine

### Geometric parameters

**Fig. 3:**
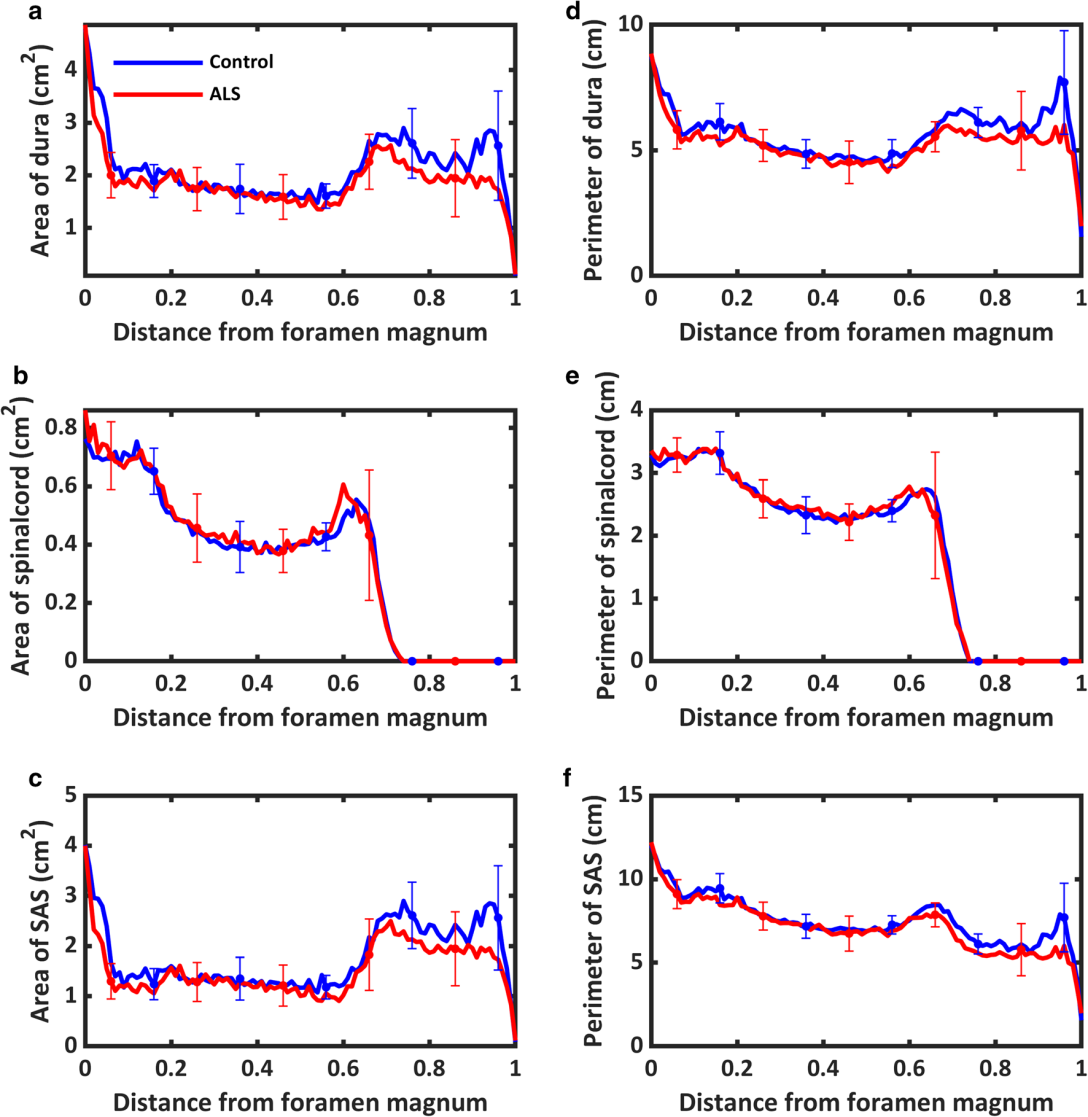
Average geometric parameter values for the ALS group (red) plotted with those of the control group (blue) in relation to distance from the FM in terms of: **a** cross-sectional area of dura, **b** cross-sectional area of spinal cord, **c** cross-sectional area of the subarachnoid space, **d** perimeter of dura, **e** perimeter of spinal cord, **f** perimeter of subarachnoid space. Parameters are plotted with respect to their normalized axial distance below the forament magnum

Visual inspection of the geometries showed a similar appearance in terms of shape between ALS patients and controls. One ALS subject (102) had a focal decrease in SSS cross-sectional area (Fig. 2). Quantitatively, cross-sectional area of the dura was found to be 11% smaller in the ALS group with the greatest difference tending to be within the dural sac region (Fig. 3a). Note, parameters are plotted with respect to their normalized axial distance below the foramen magnum. Axial SC area was nearly identical for the two groups (0.35 cm^2^, Fig. 3b and Table 1). The ALS group had a 14% smaller cross-sectional SSS area than the healthy controls (1.53 cm^2^ and 1.78 cm^2^ respectively, Fig. 3c and Table 1). Similarly, the perimeter of the dura and SSS also tended to be slightly smaller in the ALS group compared to healthy controls within the dural sac region (Fig. 3d, f). No difference was observed in the perimeter of the SC (Fig. 3e). Average volume (Table 1) of the SSS in the ALS group (97.3 cm^3^) was 10% less than the control group (108.2 cm^3^).

### CSF flow characteristics

**Fig. 4:**
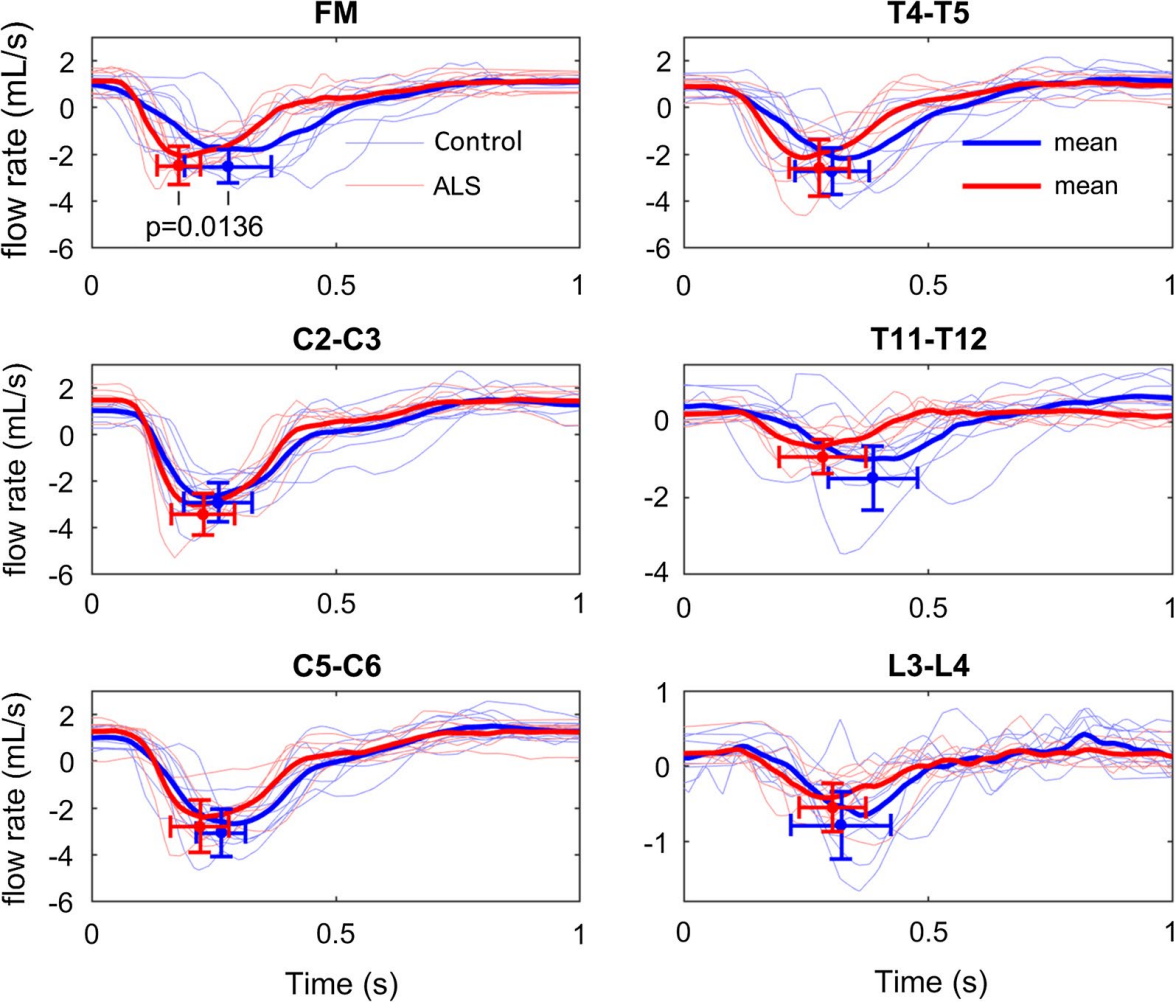
Flow rates of all subjects at the six axial locations along the spine for the ALS (red) and the control (blue) groups. The respective mean flow rate over the ALS and the control group are depicted in the bold lines. The feature points are marked at the peak systolic CSF flow with the corresponding standard deviation error bars regarding timing and flow. The FM’s peak systolic CSF flow is significantly different between the two groups regarding timing (p = 0.0136) as evaluated by the Mann–Whitney U test. Note that the y-axis scale for T11–T12 and L3–L4 are different from the other four axial locations

All flow rates from the PCMRI data measured at the FM, C2–C3, C5–C6, T4–T5, T11–T12, and L3–L4 vertebral locations are plotted for both the control (blue) and ALS (red) groups, excluding ALS case 102 in Fig. 4. Compared to the control group, peak systolic CSF flow in the ALS group was larger at C2–C3, comparable at FM, C5–C6, T4–T5 and T11–T12, and smaller at L3–L4. Only the feature point of the FM peak systolic CSF flow was significantly faster between the two groups (p = 0.0136). The maximum peak systolic CSF flow feature points assessed for every subject individually, again excluding ALS case 102 as noted above, are marked at their mean with the corresponding standard deviation error bars regarding timing and flow in Fig. 4.

**Fig. 5:**
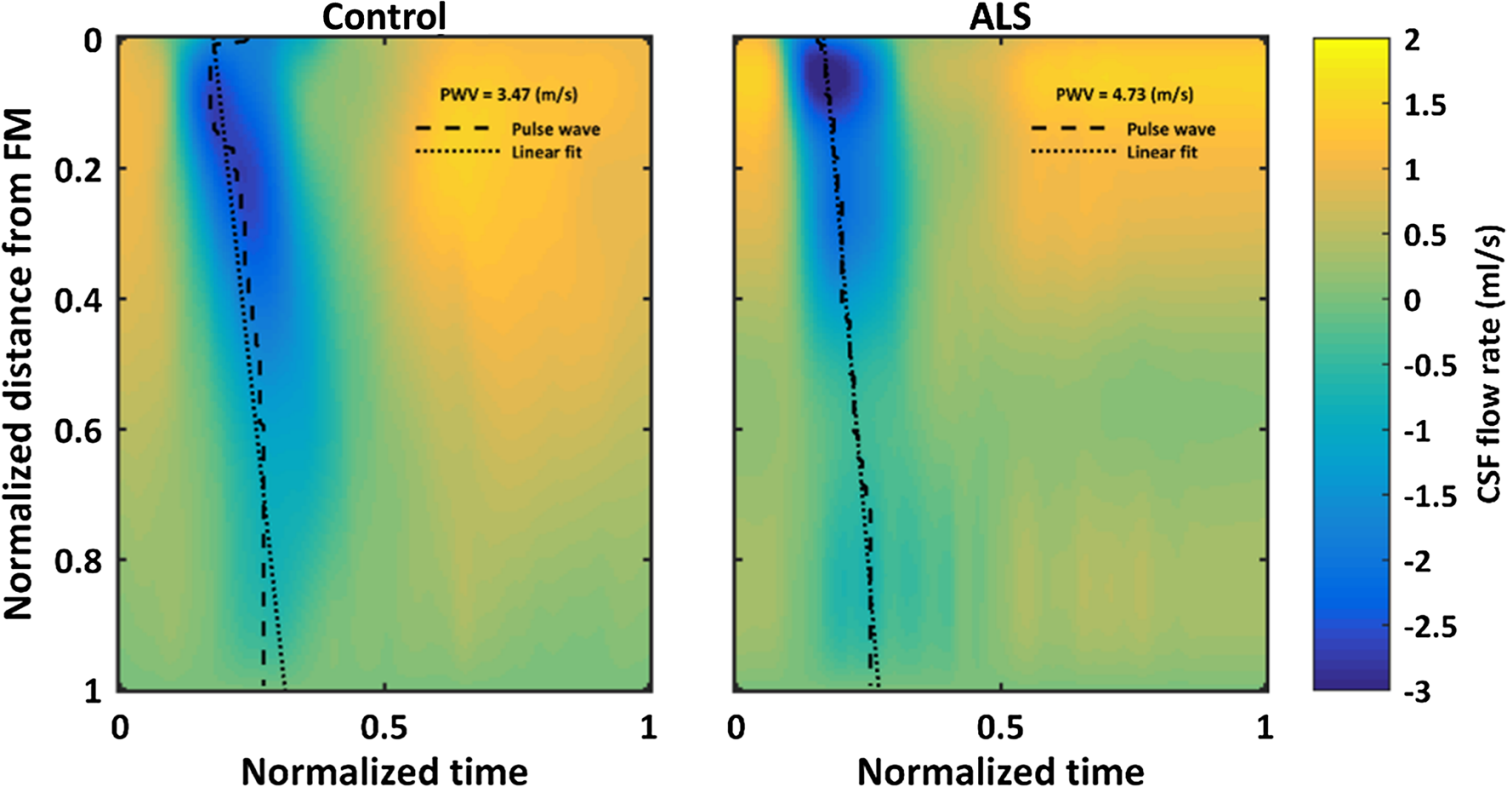
Spatial–temporal distribution of the interpolated CSF flow rates along the length of the spinal canal in the healthy control and ALS groups

Average CSF PWV along the spine was 36% faster in the ALS group (473 cm/s) compared to the control group (347 cm/s) (Table 1). The average spatial–temporal distribution of the CSF PWV for all ALS subjects excluding 102, and controls is shown in Fig. 5. Peak systolic CSF flow magnitude occurred at a normalized distance of ~ 0.05 below the FM in patients and ~ 0.2 in controls (Fig. 5).

**Fig. 6:**
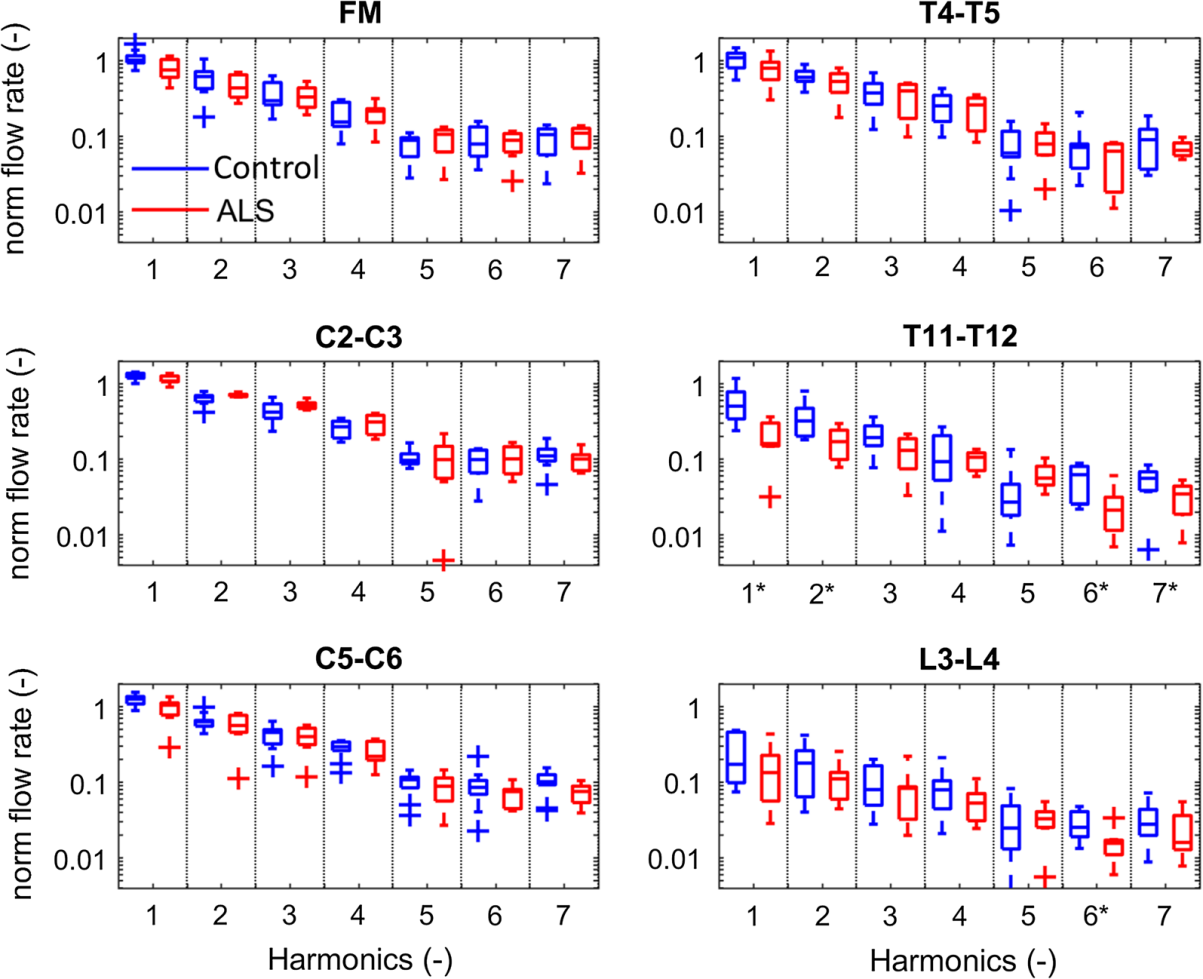
Frequency components of the normalized flow rate at the six axial locations along the spine for the ALS (red) and the healthy control (blue) groups. In each panel, the harmonics are divided by the dotted line and are presented from the first to the seventh harmonic. Significant harmonics are marked with asterisk at the respective harmonics as analyzed with the Mann–Whitney U test. The first, second, sixth, and seventh harmonic of T11–T12 are significantly different (p = 0.0031, p = 0.0136, p = 0.0097, and p = 0.0330), and at L3–L4 for the sixth harmonic (p = 0.0330)

Spectral analysis revealed that the frequency components of the normalized flow rate for the ALS (red) and the control (blue) groups are significantly different (*) for the first, second, sixth and seventh harmonic of the T11–T12 location (p = 0.0031, p = 0.0136, p = 0.0097, and p = 0.0330), and for the sixth harmonic of the L3–L4 location (p = 0.0330). The frequency components are presented from the first to the seventh harmonic in Fig. 6.

### Hydrodynamic parameters

**Fig. 7:**
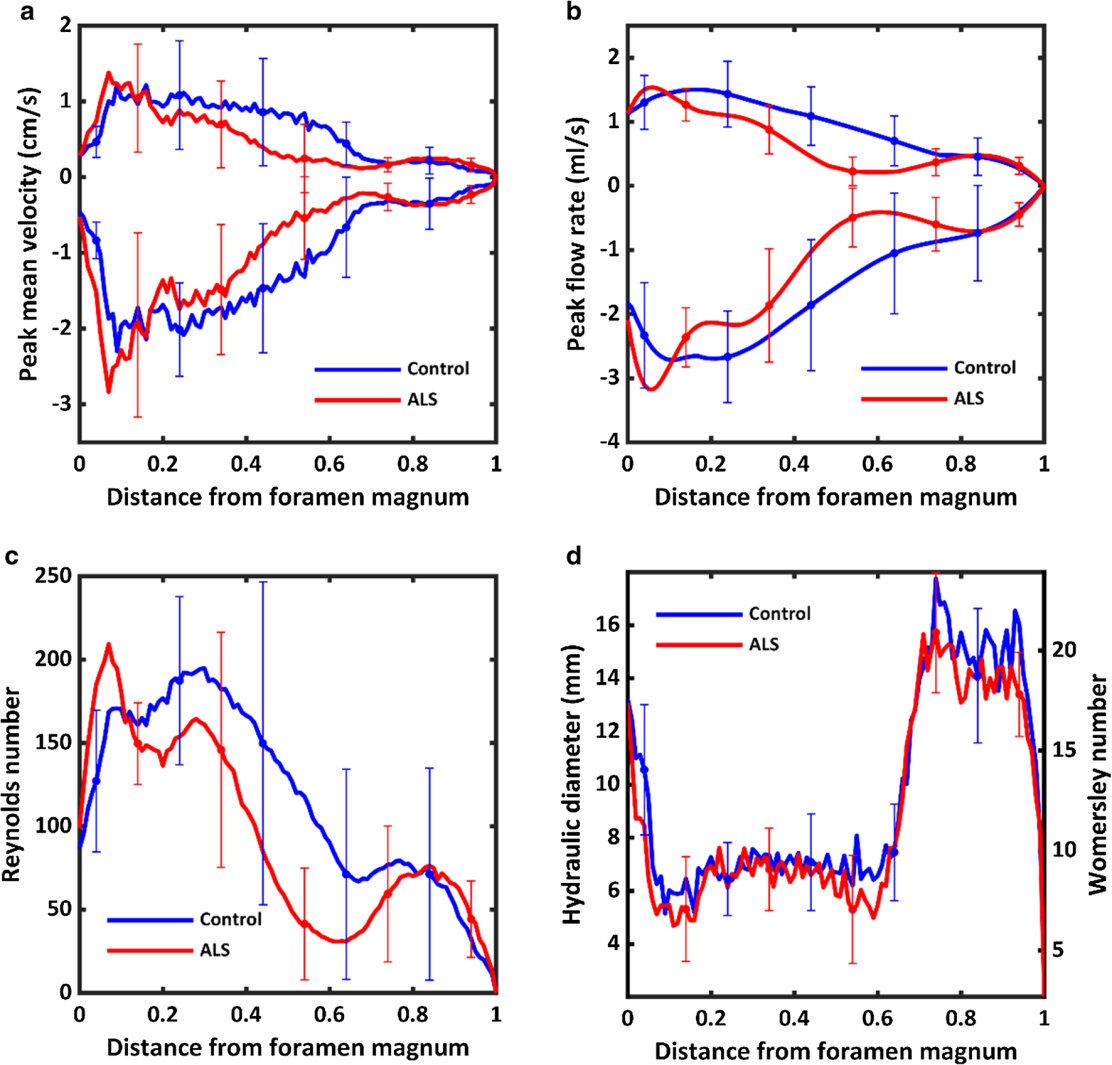
Average values for the hydrodynamic parameters quantified for ALS (red) and healthy controls (blue) along the spine in terms of: **a** peak mean velocity, **b** peak flow rate, **c** Reynolds number and **d** hydraulic diameter (left y-axis) and Womersley number (right y-axis)

All hydrodynamic parameters of interest for both the ALS group, excluding subject 102, and the control group are shown in Fig. 7 and Table 1. The systolic and diastolic CSF flow velocity along the length of the spinal cord tended to be smaller for the ALS group compared to the control group except for immediately inferior to the FM and near the dural sac (Fig. 7a). Considering both CSF geometry and velocity, the average peak systolic flow in the ALS group was 16% less than that of the control group. The average peak diastolic flow in the ALS group was 21% less than the control group (Fig. 7b). Reynolds number for the control group was 194.74 and 209.35 in the ALS group indicating laminar flow in both groups. Reynolds number was greater for the ALS group at a normalized distance of ~ 0.1 below the FM and again at ~ 0.9 (Fig. 7c). On average, $H_{D}$ in the ALS group was 7% smaller at .89 cm vs .96 cm in the control group. Womersley number behaved in a similar manner between groups for the length of the SC (Fig. 7d, right y-axis label).

## Discussion

The present study quantified geometric parameters in the spinal canal of eight people with ALS and ten healthy controls. CSF hydrodynamics were also possible to quantify and compare in seven people with ALS and ten healthy controls. This section includes the findings we feel warrant further investigation as they may have an impact on drug delivery strategies and the monitoring of ALS progression.

### Key CSF dynamics findings

Timing of peak systolic CSF velocity at the FM was significantly faster for the ALS group (p = 0.0136). Notably, one control subject had slower peak systolic CSF flow. However, because the flow amplitude was within the normal range, this subject was not considered an outlier as with ALS subject 102. Despite inclusion of this subject in the statistical analysis, timing at the FM was still found to be significantly faster in the ALS cohort. Overall, average CSF pulse wave velocity along the spine was elevated in the ALS group (473.19 cm/s) compared to the control group (347.41 cm/s) (Table 1). The average spatial–temporal distribution of the CSF PWV for all patients and controls is shown in Fig. 5. Elevated PWV may indicate an overall decrease in SSS compliance; however, arrival time of peak CSF flow was only significantly faster for the ALS group near the FM as evidence by analysis of peak CSF flow timing (Fig. 4). Peak systolic CSF flow magnitude occurred at a normalized distance of ~ 0.05 below the FM in people with ALS and ~ 0.2 in controls (Fig. 7b). CSF flow amplitude in the ALS group was only greater at the C2–C3 location and smaller at C5–C6, T11–T12, and L3–L4 than in the control group. These differences may be further indication of an overall decrease in SSS compliance with emphasis on the thoracolumbar region where changes in the spectral content of the normalized flow waveforms was most significant in the ALS group.

### Reliability of MRI based measurements of CSF hydrodynamics

Due to the sensitivity of hydrodynamic parameters to both geometry and CSF flow, it is important that the measurement technique is robust enough to produce reliable results. In a previous study by our group, Khani et al. 2019, quantified scan-rescan reliability for geometric and hydrodynamic parameters in cynomolgus monkey for which geometric parameters displayed good follow-up agreement [12]. Hydrodynamic agreement was also found to be positive although to a lesser correlative degree due to expected CSF flow variability at the 2-week follow-up scan. In cynomolgus monkeys, 174 and 123 voxels were used to quantify the average axial SSS cross-section for geometric and CSF flow analysis respectively. For the present study, 551 and 250 voxels were used to quantify the average axial SSS cross-section in human controls for geometric and CSF flow analysis respectively. In addition, the lower CSF flowrates observed in cynomolgus monkey are more difficult to measure than those found in humans. Thus, while scan-rescan reliability was not quantified in the current study, lower relative precision in cynomolgus likely result in a lower bounds of scan repeatability for identical techniques in humans. CSF PWV quantification has also been applied previously in healthy controls [30]. However, the exact test–retest reliability has not yet been quantified.

Inter-operator reliability of MRI based hydrodynamics has also been studied by our group [17]. T2-weighted MRI data collected using a 1.5 T magnet and 1.0 mm isotropic scan resolution of a healthy subject was analyzed by four operators using identical methods as the present study. The maximum coefficient of variation (CV) for cross-sectional area, peak systolic CSF flow, and Reynolds number were found to be 12.2%, 16.8% and 12.6% respectively. In the current study, CV for cross-sectional area, peak systolic CSF flow, and Reynolds number were 29.8%, 26.2% and 41.5% respectively for healthy controls and 31.4%, 25.6% and 17.8% in the ALS group. This suggests inter-individual variability in and across groups is above the level explained by inter-operator variability alone.

Additionally, in vitro studies have been performed by our group to quantify the reliability of MRI for CSF geometric and flow measurements. Yildiz et al. 2017, found a CV of 4.8% for peak CSF flow at a single location [31] and Thyagaraj et al. 2017, found a an average CV of 8% for peak CSF flow across several locations [32]. Geometric reconstruction was also analyzed by Thyagaraj et al. 2017, and found to be in good agreement with the digital. STL geometry used to create the flow phantom; however, some systematic over estimation in reconstructed geometric variables was noted [32]. Overall, these observations provide further support for the robustness of the current method to be sufficient for observation of differences in and among groups.

### Comparison of geometric results to previous studies

Review of CSF volume within the SSS was provided by Sass et al. 2017, and averaged 80.0 cm^3^ for all studies reporting values of the complete SSS [26]. In our study, CSF volume in the SSS averaged 97.3 cm^3^ for the ALS group, 10% less than the control group at 108.2 cm^3^. Qualitatively, this difference was not evident based on visual inspection, which revealed a similar degree of geometric variability between both ALS and control cohorts (Fig. 2). Volumetric calculations are sensitive to small changes in segmentation accuracy which is itself dependent on image resolution. While somewhat higher, our values for SSS volume are well within the range of reported values. Furthermore, when compared against average SSS volume from the small number of manual segmentations of healthy subjects, agreement is notable with those studies also averaging 108.2 cm^3^. Finally, trends in axial distribution of cross-sectional areas within our study for both spinal cord and dura show good similarity to Loth et al. 2001 [28], with peaks in SSS area located at the FM and lumbar enlargement (Fig. 3a, b). While T2-weighted MRI data for the full cranial volume was captured as part of the scanning protocol, we did not capture CSF flow around the brain or aqueduct of Sylvius. This was due to consideration of patient time in the scanner approaching 1 h, and especially the sensitivity of the ALS group to prolonged supine position.

### MRI based ALS biomarkers investigated in previous studies

The ideal ALS biomarker would have sufficient sensitivity and specificity for the syndrome, predict regional involvement and symptom spread, ability to differentiate clinical phenotypes, and be based on easily accessible and affordable technology [33]. CSF-based biomarkers such as CSF hydrodynamics show promise because of the CSF’s innate proximity to the location of disease involvement. Recent studies have shown that there is rapid exchange of CSF with the brain parenchyma during sleep acting to wash the brain [34, 35] as well as a possible glymphatic mechanism which removes waste products from the CSF [36]. Therefore, disruption of normal CSF dynamics could, in principle, play a role in the progression of degenerative CNS disease.

While many MR imaging techniques have been used to investigate ALS biomarkers and disease progression [33, 37], the primary focus has been the CNS tissue, rather than the CSF surrounding that tissue. Conversely, research evaluating ALS molecular biomarkers in the CSF has been well established for many decades with a large number of possible disease biomarkers identified often in elevated levels. Disruption of the BBB has been implicated in a number of neurodegenerative diseases and is also possible to study through MRI imaging [38, 39]. Coupling this understanding with CSF distribution and flow dynamics may provide further insight and predictors into the rate or mechanisms of the disease progression.

Progressive death of motor neurons in ALS leads to observable changes in the brain and spinal cord as highlighted by El Mendili et al. 2019 [40]. Longitudinal studies have established a connection between cervical spinal cord atrophy and functional decline in ALS patients [41–43]. Additionally, reviews on the utility of various imaging techniques for visualizing changes in CNS have discussed a potential use in the diagnosis and tracking of ALS [33, 44, 45]. In particular, a study Sato et al. 2012 [24], utilized PCMRI to evaluate CSF pulsatility in 40 subjects with motor neuron disease as well as 14 healthy controls. An average peak systolic CSF velocity of − 6.0 cm/s, − 5.4 cm/s and − 3.3 cm/s for was found for combined motor neuron disease, elderly controls and young controls, respectively. Furthermore, no significant difference of CSF pulse wave timing at C5 was found between the study groups. Flow velocities reported in the above study are somewhat higher than those found by our group (Fig. 7a). However, it should be noted that these measurements were taken at two small regions of interest bilaterally adjacent to the spinal cord. Our group has previously shown that CSF flow distribution is variable across the spinal canal cross-section and can include localized flow “jets”  [13]. We therefore considered the average CSF velocity across the entire SSS cross-section which includes regions of both high and low flow rates. Finally, regarding CSF pulse wave timing, the aforementioned study by Sato and colleagues is consistent with our findings at the analogous location of C5/C6 finding no statistical difference. Importantly, our study contributes a novel analysis of hydrodynamics across the entire SSS geometry based on CSF flow measurements across the full SSS cross-section at multiple locations.

### Potential implications for intrathecal ALS therapeutics

The proximity of CSF with the CNS tissue makes it a potential route for ALS treatment using intrathecal drug delivery. While IT drug delivery is a growing field, due to a gap in foundational knowledge and higher associated risks, IT devices and therapeutics are not common clinically [46]. Intrathecal delivery baclofen is among the only IT therapy regularly prescribed for ALS where systemic side-effects are dose limiting and pain due to severe spasticity cannot be effectively managed by other means [47–49]. Ultimately, intrathecal baclofen is not a disease modifying treatment and symptomatic benefits need to be carefully titrated against retention of beneficial muscle tone [50].

At present, only two approved therapies, Riluzole and Edaravone, are shown to potentially produce modest delay in ALS progression, however neither is administered intrathecally in humans [51]. Intrathecal delivery of Riluzole has been investigated in both Gottingen minipigs and canine models [52, 53] with both studies demonstrating higher Riluzole levels in the CNS while limiting the systemic dose that may lead to off-target side-effects. Additionally, gene therapy and therapies involving trophic factors to stimulate dying neurons [54, 55] have shown promise in rodent models. Other animal studies have also shown that human stem cells administered intrathecally delay the onset of symptoms and prolong survival in ALS transgenic mice [56]. The mechanism by which preservation, and in some cases, regeneration of motor neurons occurs appears to be due to production of growth factors and other neuroprotective compounds that can be found in CSF [56, 57]. Additionally, alteration of the neurotoxic environment observed in ALS is another potential target for treating this devastating disease [58–60] and could be effected via CSF filtration [10, 11]. This could potentially improve the survivability of transplanted stem cells and improve effectiveness of other IT treatments. Overall, most potential IT therapeutic approaches for ALS remain experimental and are often based on specific induced forms of the disease in animal models. While the reduction of the above findings to clinical application is yet to be seen, the potential for CSF hydrodynamics to inform the design and application of new IT therapeutics and devices is still clear.

The observed differences in systolic and diastolic flow, volume of the SSS, and CSF geometric and hydrodynamic properties are important for development of accurate models for IT drug administration and manipulation of the spinal CSF environment. Several studies have used MRI data (frequently of healthy individuals) to derive in vitro and computer-generated models for analyzing dispersion of compounds in the SSS and pulsatile flow is consistently indicated as one of the major contributors to CSF mixing [12, 61–64]. One of our subjects (102) exhibited nearly zero CSF flow while others had more modest decreases compared to controls. This subject also had a local SSS restriction in the cervical spine that was present around the entire circumference of the spinal cord as well as suspected redundant nerve root syndrome in the lumbar spine (Fig. 2). While local variability in the cross-sectional area of the SSS was noticeable in both groups, with the exception of ALS subject 102, there were no visible features which could consistently be correlated with CSF dynamics (Fig. 2). In the case of ALS subject 102, it is likely that the observed restrictions decreased the CSF pulsation along the entire spine. Because the rate of diffusion within spinal CSF is many orders of magnitude slower than in advective mixing, this type of focal restriction could have an important impact in context of IT solute transport [65].

### Limitations

Several limitations exist in our study. Findings for both groups were based on a relatively small sample. Rarity and variability of the disease combined with careful screening against the ability to undergo a protracted MRI-scan presented a distinct challenge in finding participants on a regional level. Secondly, our control group was not ideally matched against our ALS group and both groups included subjects with confounding conditions. While these factors negatively impacted the statistical power of the results in this study, it is straightforward to expand this data set in future with additional subjects. Furthermore, a longitudinal study would also allow comparison of any observations with disease progression.

Lastly, while raw data was collected at a relatively high resolution, micro anatomy such as nerve roots, and denticulate ligaments were not possible to visualize. While important for specific transport dynamics, nerve roots did not have a large effect on unsteady CSF velocities as show in our previous computational study [27]. Similarly, flow measurements were made at only a few locations along the spinal canal and it was not possible to capture transient flow phenomena due to phase averaging. This was primarily a practical limitation of scan duration which was already at ~ 45 min.

## Conclusion

This study characterized CSF flow dynamics alongside geometric parameters in humans with ALS as well healthy controls. We found significant differences in peak systolic CSF flow timing at the FM, as well significant differences in the spectral content of CSF waveforms between ALS and control cohorts. More modest and non-significant differences in the CSF dynamics of our ALS group showed reduced CSF flow magnitude and increased PWV. While our study lacks sufficient power to draw definite conclusions regarding the differences we observed, we believe they deserve further investigation because of their potential importance related to intrathecal solute transport. In particular, a growing interest in IT drug delivery and the possible connection of trophic and neurotoxic factors in the CSF with disease progression warrant further study of CSF dynamics in the disease state. With the high degree of heterogeneity that exists among ALS cases it may be beneficial to conduct larger, longitudinal studies to determine how changes in CSF flow correlate with disease progression. This may contribute to the understanding of the pathologic progression of ALS, particularly if the onset of a neurotoxic CSF environment and breakdown of CSF flow were to coincide.

## Data Availability

The data that support the findings of this study are openly available for request from the corresponding author.

## Abbreviations

3D: three-dimensional
ALS: amyotrophic lateral sclerosis
BBB: blood brain barrier
CNS: central nervous system
CSF: cerebrospinal fluid
CV: coefficient of variation
DM: dura matter
FFT: fast Fourier transform
FM: foramen magnum
FOV: field of view
IT: intrathecal
MR: magnetic resonance
MRI: magnetic resonance imaging
PCMRI: phase contrast magnetic resonance imaging
PWV: pulse wave velocity
SC: spinal cord
SPACE: sampling perfection with application optimized contrasts using different flip angle evolution
SSS: spinal subarachnoid space
TE: echo time
TR: repetition time

## References

[1] Schymick JC, Talbot K, Traynor BJ (2007). Genetics of sporadic amyotrophic lateral sclerosis. Hum Mol Genet.

[2] Alsultan AA, Waller R, Heath PR, Kirby J (2016). The genetics of amyotrophic lateral sclerosis: current insights. Degener Neurol Neuromuscul Dis.

[3] Ravits JM, La Spada AR (2009). ALS motor phenotype heterogeneity, focality, and spread deconstructing motor neuron degeneration. Neurology.

[4] Mehta P, Antao V, Kaye W, Sanchez M, Williamson D, Bryan L, Muravov O, Muravov K (2014). Prevalence of amyotrophic lateral sclerosis—United States, 2010–2011. MMWR Suppl.

[5] McCombe PA, Henderson RD (2010). Effects of gender in amyotrophic lateral sclerosis. Gend Med.

[6] Van Damme P, Robberecht W (2014). Developments in treatments for amyotrophic lateral sclerosis via intracerebroventricular or intrathecal delivery. Expert Opin Investig Drugs.

[7] Kiernan MC, Vucic S, Cheah BC, Turner MR, Eisen A, Hardiman O (2011). Amyotrophic lateral sclerosis. Lancet.

[8] Smilnak GJ, Charalambous LT, Cutshaw D, Premji AM, Giamberardino CD, Ballard CG (2018). Novel treatment of cryptococcal meningitis via neurapheresis therapy. J Infect Dis.

[9] Blackburn SL, Swisher CB, Grande AW, Rubi A, Verbick LZ, McCabe A (2019). Novel dual lumen catheter and filtration device for removal of subarachnoid hemorrhage: first case report. Oper Neurosurg.

[10] Finsterer J, Mamoli B (1999). Liquorpheresis (CSF filtration) in familial amyotrophic lateral sclerosis. Spinal Cord.

[11] Finsterer J, Mamoli B (1999). Cerebrospinal fluid filtration in amyotrophic lateral sclerosis. Eur J Neurol.

[12] Khani M, Lawrence BJ, Sass LR, Gibbs CP, Pluid JJ, Oshinski JN (2019). Characterization of intrathecal cerebrospinal fluid geometry and dynamics in cynomolgus monkeys (*Macaca fascicularis*) by magnetic resonance imaging. PLoS ONE.

[13] Khani M, Xing T, Gibbs C, Oshinski JN, Stewart GR, Zeller JR (2017). Nonuniform moving boundary method for computational fluid dynamics simulation of intrathecal cerebrospinal flow distribution in a cynomolgus monkey. J Biomech Eng.

[14] Tangen KM, Hsu Y, Zhu DC, Linninger AA (2015). CNS wide simulation of flow resistance and drug transport due to spinal microanatomy. J Biomech.

[15] Martin BA, Kalata W, Shaffer N, Fischer P, Luciano M, Loth F (2013). Hydrodynamic and longitudinal impedance analysis of cerebrospinal fluid dynamics at the craniovertebral junction in type I Chiari malformation. PLoS ONE.

[16] Shaffer N, Martin BA, Rocque B, Madura C, Wieben O, Iskandar BJ (2014). Cerebrospinal fluid flow impedance is elevated in type I Chiari malformation. J Biomech Eng Trans ASME.

[17] Martin BA, Yiallourou TI, Pahlavian SH, Thyagaraj S, Bunck AC, Loth F (2016). Inter-operator reliability of magnetic resonance image-based computational fluid dynamics prediction of cerebrospinal fluid motion in the cervical spine. Ann Biomed Eng.

[18] Lloyd RA, Fletcher DF, Clarke EC, Bilston LE (2017). Chiari malformation may increase perivascular cerebrospinal fluid flow into the spinal cord: a subject-specific computational modelling study. J Biomech.

[19] Linge SO, Haughton V, Lovgren AE, Mardal KA, Helgeland A, Langtangen HP (2011). Effect of tonsillar herniation on cyclic CSF flow studied with computational flow analysis. Am J Neuroradiol.

[20] Clarke EC, Fletcher DF, Stoodley MA, Bilston LE (2013). Computational fluid dynamics modelling of cerebrospinal fluid pressure in Chiari malformation and syringomyelia. J Biomech.

[21] Bunck AC, Kroeger JR, Juettner A, Brentrup A, Fiedler B, Crelier GR (2012). Magnetic resonance 4D flow analysis of cerebrospinal fluid dynamics in Chiari I malformation with and without syringomyelia. Eur Radiol.

[22] Cheng S, Stoodley MA, Wong J, Hemley S, Fletcher DF, Bilston LE (2012). The presence of arachnoiditis affects the characteristics of CSF flow in the spinal subarachnoid space: a modelling study. J Biomech.

[23] Nowoslawska E, Gwizdala D, Baranska D, Grzelak P, Podgorski M, Zakrzewski K (2018). The oscillatory flow of the cerebrospinal fluid in the Sylvian aqueduct and the prepontine cistern measured with phase contrast MRI in children with hydrocephalus-a preliminary report. Childs Nerv Syst.

[24] Sato K, Morimoto N, Matsuura T, Ohta Y, Tsunoda M, Ikeda Y (2012). CSF flow dynamics in motor neuron disease. Neurol Res.

[25] Martin BA, Kalata W, Loth F, Royston TJ, Oshinski JN (2005). Syringomyelia hydrodynamics: an in vitro study based on in vivo measurements. J Biomech Eng Trans ASME.

[26] Sass LR, Khani M, Natividad GC, Tubbs RS, Baledent O, Martin BA (2017). A 3D subject-specific model of the spinal subarachnoid space with anatomically realistic ventral and dorsal spinal cord nerve rootlets. Fluids Barriers CNS.

[27] Khani M, Sass L, Xing T, Sharp MK, Balédent O, Martin B (2018). Anthropomorphic model of intrathecal cerebrospinal fluid dynamics within the spinal subarachnoid space: spinal cord nerve roots increase steady-streaming. J Biomech Eng.

[28] Loth F, Yardimci MA, Alperin N (2001). Hydrodynamic modeling of cerebrospinal fluid motion within the spinal cavity. J Biomech Eng.

[29] Gehlen M, Kurtcuoglu V, Schmid Daners M (2017). Is posture-related craniospinal compliance shift caused by jugular vein collapse? A theoretical analysis. Fluids Barriers CNS.

[30] Martin BA, Loth F, Kalata W, Oshinski JN, editors. MR measurement of pulse wave velocity in the spinal canal. In: Proceedings of the ASME summer bioengineering conference, SBC2008; 2009.

[31] Yildiz S, Thyagaraj S, Jin N, Zhong X, Heidari Pahlavian S, Martin BA (2017). Quantifying the influence of respiration and cardiac pulsations on cerebrospinal fluid dynamics using real-time phase-contrast MRI. J Magn Reson Imaging.

[32] Thyagaraj S, Pahlavian SH, Sass LR, Loth F, Vatani M, Choi JW (2017). An MRI-compatible hydrodynamic simulator of cerebrospinal fluid motion in the cervical spine. IEEE Trans Biomed Eng.

[33] Turner MR, Kiernan MC, Leigh PN, Talbot K (2009). Biomarkers in amyotrophic lateral sclerosis. Lancet Neurol.

[34] Xie LL, Kang HY, Xu QW, Chen MJ, Liao YH, Thiyagarajan M (2013). Sleep drives metabolite clearance from the adult brain. Science.

[35] O’Donnell J, Ding F, Nedergaard M (2015). Distinct functional states of astrocytes during sleep and wakefulness: is norepinephrine the master regulator?. Curr Sleep Med Rep.

[36] Ringstad G, Vatnehol SAS, Eide PK (2017). Glymphatic MRI in idiopathic normal pressure hydrocephalus. Brain.

[37] Castellano A, Papinutto N, Cadioli M, Brugnara G, Iadanza A, Scigliuolo G (2016). Quantitative MRI of the spinal cord and brain in adrenomyeloneuropathy: in vivo assessment of structural changes. Brain.

[38] Sweeney MD, Sagare AP, Zlokovic BV (2018). Blood-brain barrier breakdown in Alzheimer disease and other neurodegenerative disorders. Nat Rev Neurol.

[39] Kermode AG, Thompson AJ, Tofts P, Macmanus DG, Kendall BE, Kingsley DPE (1990). Breakdown of the blood-brain-barrier precedes symptoms and other MRI signs of new lesions in multiple-sclerosis—pathogenetic and clinical implications. Brain.

[40] El Mendili MM, Querin G, Bede P, Pradat PF (2019). Spinal cord imaging in amyotrophic lateral sclerosis: historical concepts-novel techniques. Front Neurol.

[41] Paquin ME, El Mendili MM, Gros C, Dupont SM, Cohen-Adad J, Pradat PF (2018). Spinal cord gray matter atrophy in amyotrophic lateral sclerosis. AJNR Am J Neuroradiol.

[42] El Mendili MM, Cohen-Adad J, Pelegrini-Issac M, Rossignol S, Morizot-Koutlidis R, Marchand-Pauvert V (2014). Multi-parametric spinal cord MRI as potential progression marker in amyotrophic lateral sclerosis. PLoS ONE.

[43] Branco LM, De Albuquerque M, De Andrade HM, Bergo FP, Nucci A, Franca MC (2014). Spinal cord atrophy correlates with disease duration and severity in amyotrophic lateral sclerosis. Amyotroph Lateral Scler Frontotemporal Degener.

[44] Turner MR, Benatar M (2015). Ensuring continued progress in biomarkers for amyotrophic lateral sclerosis. Muscle Nerve.

[45] Agosta F, Chio A, Cosottini M, De Stefano N, Falini A, Mascalchi M (2010). The present and the future of neuroimaging in amyotrophic lateral sclerosis. Am J Neuroradiol.

[46] Nagel SJ, Reddy CG, Frizon LA, Holland MT, Machado AG, Gillies GT (2018). Intrathecal therapeutics: device design, access methods, and complication mitigation. Neuromodulation.

[47] Sadiq SA, Wang GC (2006). Long-term intrathecal baclofen therapy in ambulatory patients with spasticity. J Neurol.

[48] McClelland S, Bethoux FA, Boulis NM, Sutliff MH, Stough DK, Schwetz KM (2008). Intrathecal baclofen for spasticity-related pain in amyotrophic lateral sclerosis: efficacy and factors associated with pain relief. Muscle Nerve.

[49] Saulino M, Ivanhoe CB, McGuire JR, Ridley B, Shilt JS, Boster AL (2016). Best practices for intrathecal baclofen therapy: patient selection. Neuromodulation.

[50] Paganoni S, Karam C, Joyce N, Bedlack R, Carter GT (2015). Comprehensive rehabilitative care across the spectrum of amyotrophic lateral sclerosis. NeuroRehabilitation.

[51] Dash RP, Babu RJ, Srinivas NR (2018). Two decades-long journey from Riluzole to Edaravone: revisiting the clinical pharmacokinetics of the only two amyotrophic lateral sclerosis therapeutics. Clin Pharmacokinet.

[52] Gutierrez J, Federici T, Peterson B, Bartus R, Betourne A, Boulis NM (2016). 321 Development of intrathecal riluzole: a new route of administration for the treatment of amyotrophic lateral sclerosis patients. Neurosurgery.

[53] Boulis NM (2019). Intrathecal riluzole for the treatment of patients with amyotrophic lateral sclerosis. Neurosurgery.

[54] Watanabe Y, Kazuki Y, Kazuki K, Ebiki M, Nakanishi M, Nakamura K (2015). Use of a human artificial chromosome for delivering trophic factors in a rodent model of amyotrophic lateral sclerosis. Mol Ther Nucleic Acids.

[55] Deepa P, Shahani N, Alladi PA, Vijayalakshmi K, Sathyaprabha TN, Nalini A (2011). Down regulation of trophic factors in neonatal rat spinal cord after administration of cerebrospinal fluid from sporadic amyotrophic lateral sclerosis patients. J Neural Transm.

[56] Hwang DH, Lee HJ, Park IH, Seok JI, Kim BG, Joo IS (2009). Intrathecal transplantation of human neural stem cells overexpressing VEGF provide behavioral improvement, disease onset delay and survival extension in transgenic ALS mice. Gene Ther.

[57] Kerr DA, Llado J, Shamblott MJ, Maragakis NJ, Irani DN, Crawford TO (2003). Human embryonic germ cell derivatives facilitate motor recovery of rats with diffuse motor neuron injury. J Neurosci.

[58] Lindvall O, Kokaia Z (2010). Stem cells in human neurodegenerative disorders–time for clinical translation?. J Clin Invest.

[59] Henkel JS, Beers DR, Zhao W, Appel SH (2009). Microglia in ALS: the good, the bad, and the resting. J Neuroimmune Pharmacol.

[60] Shaw PJ (2002). Toxicity of CSF in motor neurone disease: a potential route to neuroprotection. Brain.

[61] Stockman HW (2007). Effect of anatomical fine structure on the dispersion of solutes in the spinal subarachnoid space. J Biomech Eng.

[62] Kuttler A, Dimke T, Kern S, Helmlinger G, Stanski D, Finelli LA (2010). Understanding pharmacokinetics using realistic computational models of fluid dynamics: biosimulation of drug distribution within the CSF space for intrathecal drugs. J Pharmacokinet Pharmacodyn.

[63] Hsu Y, Hettiarachchi HD, Zhu DC, Linninger AA (2012). The frequency and magnitude of cerebrospinal fluid pulsations influence intrathecal drug distribution: key factors for interpatient variability. Anesth Analg.

[64] Hettiarachchi HDM, Hsu Y, Harris TJ, Linninger AA (2011). The effect of pulsatile flow on intrathecal drug delivery in the spinal canal. Ann Biomed Eng.

[65] Keith Sharp M, Carare RO, Martin BA (2019). Dispersion in porous media in oscillatory flow between flat plates: applications to intrathecal, periarterial and paraarterial solute transport in the central nervous system. Fluids Barriers CNS.

